# Diagnosis of lethal or prenatal‐onset autosomal recessive disorders by parental exome sequencing

**DOI:** 10.1002/pd.5175

**Published:** 2017-12-03

**Authors:** Karen L. Stals, Matthew Wakeling, Júlia Baptista, Richard Caswell, Andrew Parrish, Julia Rankin, Carolyn Tysoe, Garan Jones, Adam C. Gunning, Hana Lango Allen, Lisa Bradley, Angela F. Brady, Helena Carley, Jenny Carmichael, Bruce Castle, Deirdre Cilliers, Helen Cox, Charu Deshpande, Abhijit Dixit, Jacqueline Eason, Frances Elmslie, Andrew E. Fry, Alan Fryer, Muriel Holder, Tessa Homfray, Emma Kivuva, Victoria McKay, Ruth Newbury‐Ecob, Michael Parker, Ravi Savarirayan, Claire Searle, Nora Shannon, Deborah Shears, Sarah Smithson, Ellen Thomas, Peter D. Turnpenny, Vinod Varghese, Pradeep Vasudevan, Emma Wakeling, Emma L. Baple, Sian Ellard

**Affiliations:** ^1^ Molecular Genetics Department Royal Devon and Exeter NHS Foundation Trust Exeter UK; ^2^ Institute of Biomedical and Clinical Science University of Exeter Medical School Exeter UK; ^3^ Clinical Genetics Department Royal Devon and Exeter NHS Foundation Trust Exeter UK; ^4^ Department of Clinical Genetics Our Lady's Children's Hospital Dublin Ireland; ^5^ North West Thames Regional Genetics Service London North West Healthcare NHS Trust Harrow UK; ^6^ Guy's Regional Genetics Service Guy's and St Thomas' NHS Foundation Trust London UK; ^7^ Oxford Regional Clinical Genetics Service Northampton General Hospital Northampton UK; ^8^ Oxford Centre for Genomic Medicine Oxford University Hospitals NHS Foundation Trust Oxford UK; ^9^ West Midlands Medical Genetics Department Birmingham Women's Hospital Birmingham UK; ^10^ Department of Clinical Genetics, City Campus Nottingham University Hospitals NHS Trust Nottingham UK; ^11^ South West Thames Regional Genetics Service St George's University Hospitals NHS Foundation Trust London UK; ^12^ Institute of Medical Genetics University Hospital of Wales Cardiff UK; ^13^ Department of Clinical Genetics Liverpool Women's NHS Foundation Trust Liverpool UK; ^14^ Department of Clinical Genetics University Hospital Bristol Bristol UK; ^15^ Sheffield Clinical Genetics Service Sheffield Children's Hospital Sheffield UK; ^16^ Victorian Clinical Genetics Services Murdoch Children's Research Institute Melbourne Vic Australia; ^17^ Leicester Clinical Genetics, Women's and Children's Services Leicester Royal Infirmary Leicester UK; ^18^ Wessex Clinical Genetics Service University Hospital Southampton NHS Foundation Trust Southampton UK

## Abstract

**Objective:**

Rare genetic disorders resulting in prenatal or neonatal death are genetically heterogeneous, but testing is often limited by the availability of fetal DNA, leaving couples without a potential prenatal test for future pregnancies. We describe our novel strategy of exome sequencing parental DNA samples to diagnose recessive monogenic disorders in an audit of the first 50 couples referred.

**Method:**

Exome sequencing was carried out in a consecutive series of 50 couples who had 1 or more pregnancies affected with a lethal or prenatal‐onset disorder. In all cases, there was insufficient DNA for exome sequencing of the affected fetus. Heterozygous rare variants (MAF < 0.001) in the same gene in both parents were selected for analysis. Likely, disease‐causing variants were tested in fetal DNA to confirm co‐segregation.

**Results:**

Parental exome analysis identified heterozygous pathogenic (or likely pathogenic) variants in 24 different genes in 26/50 couples (52%). Where 2 or more fetuses were affected, a genetic diagnosis was obtained in 18/29 cases (62%). In most cases, the clinical features were typical of the disorder, but in others, they result from a hypomorphic variant or represent the most severe form of a variable phenotypic spectrum.

**Conclusion:**

We conclude that exome sequencing of parental samples is a powerful strategy with high clinical utility for the genetic diagnosis of lethal or prenatal‐onset recessive disorders. © 2017 The Authors Prenatal Diagnosis published by John Wiley & Sons Ltd.

## Abstract 2

What's already known about this topic?

- Exome sequencing is used routinely for postnatal diagnosis of rare disorders with a diagnostic yield of 20 to 40%.
- Insufficient quantity or quality of DNA restricts the use of exome sequencing for diagnosing lethal fetal disorders.
- Couples are counselled for a likely 25% recurrence risk, but without a genetic diagnosis, no molecular prenatal test is possible.
- A parental exome sequencing strategy has been applied successfully in a small number of couples.

What does this study add?

- We show that exome sequencing of parental DNA samples is an effective way to diagnose lethal or prenatal‐onset disorders with a diagnostic yield of 52% in an audit of 50 consecutive cases.
- Testing can be carried out in the prenatal period to guide management of an ongoing pregnancy or for use in subsequent pregnancies to allow couples the option of a prenatal or preimplantation genetic test.

## Introduction

Whole exome sequencing (WES) in the postnatal setting has a diagnostic yield of 25 to 37%.^1^, ^2^, ^3^, ^4^ Trio exome sequencing is often considered to be the strategy of choice as this can identify both inherited and de novo variants but is also the most expensive and relies on availability of large quantities of good quality DNA samples for the affected child and both parents. Trio exome analysis is often not possible for couples with single or multiple pregnancies affected with rare lethal disorders due to limited DNA quantity and/or quality of fetal DNA (if only formalin‐fixed paraffin‐embedded tissue is available), leaving these couples without a diagnosis and limited reproductive choices.^5^ Several studies have demonstrated the utility of exome sequence analysis for fetuses where DNA quantity is not limited.^6^, ^7^ In addition to DNA availability, other factors make diagnosis difficult in lethal fetal disorders, including the large number of potential genes, phenotypic variability, and the difficulty in accurately phenotyping a mid‐gestation fetus. Single molecular tests may be guided by the limited phenotyping available, but testing single genes in a step‐wise manner may exhaust the little fetal material that may be available. This approach often fails to make a diagnosis, and ultrasound diagnosis in the second trimester is the only potential option for many couples, with the consequence of prolonged uncertainty regarding the pregnancy outcome and the possibility of a late gestation termination.^5^, ^6^ Where multiple pregnancies are affected, the disorders are very likely to be recessive, and a recurrence risk of at least 25% is given for each future pregnancy but with no prenatal test available for future pregnancies.

We previously described a strategy utilizing parental exome sequencing and the application of a set of filtering criteria as an alternative method to identify potentially pathogenic variants in shared genes in unrelated, unaffected parents.^5^ This strategy saves precious fetal DNA as only a small amount is used for co‐segregation studies. Identification of a genetic diagnosis will enable prenatal diagnosis or preimplantation genetic diagnosis in subsequent pregnancies. This parental sequencing strategy has been applied in a recent study of prenatal‐onset cases where the authors describe the approach as “molecular autopsy by proxy” and report a high diagnostic yield.^7^ Following our early work,^5^ we introduced a diagnostic service, and the current study is an audit of the first 50 couples referred for parental exome sequencing. The couples had at least 1 fetus affected with severe fetal malformations or underwent termination of a pregnancy for a severely disabling disorder. In contrast to our previous report, in this large case series, the analysis was not limited to unrelated couples or couples with 2 or more affected pregnancies. Variants were shortlisted for evaluation by using a bioinformatics pipeline to identify potentially recessive likely pathogenic variants in established disease‐causing genes that were consistent with the clinical phenotype. Co‐segregation studies were carried out by using the stored DNA samples from the affected fetus(es). Here, we highlight the effectiveness of this strategy and the benefits of integrating exome sequencing for lethal prenatal disorders into the diagnostic pathway for these couples.

## Methods

### Subjects

The case series was composed of 50 couples who had had 1 or more fetal/neonatal losses or terminated pregnancies based on the presence of malformations detected by ultrasound scanning. Of these, 29 had 2 or more affected pregnancies. Known consanguinity was reported in 11 couples. Two couples had offspring that died at birth but were tested with this method due to limited DNA availability from the affected babies (cases 31 and 41). The couples were referred for exome sequencing from centers across the UK and Ireland. All patients provided informed consent for testing to identify a genetic cause of the disorder affecting their pregnancies. The clinical description provided for each case by the referring clinician was converted to human phenotype ontology (HPO) terms by using the ontobee browser (http://www.ontobee.org/) and HPO browser (http://human‐phenotype‐ontology.github.io/tools.html). The HPO terms for the case series are shown in Table S1.

### Exome library preparation and sequencing

Genomic DNA samples were quantified according to the manufacturer's instructions on the Qubit fluorimeter (Thermo Fisher Scientific, Massachusetts, USA) to determine that the minimum quantity of DNA required, 3000 ng, was available. The samples were fragmented by using the Bioruptor (Diagenode, Liège, Belgium) and indexed adaptors ligated before hybridization with the Agilent SureSelect All Exon capture kit (v4, v5, or v6) or Agilent SureSelect Focused exome kit (Santa Clara, CA, USA). Paired‐end 100‐bp reads were sequenced on a HiSeq 2500 (Illumina, San Diego, CA, USA) by using either the standard or the rapid run mode or paired‐end 150‐bp reads on the NextSeq500 (Illumina, San Diego, CA, USA) by using either a mid or high output flow cell. Approximately 12 whole exomes can be run per flow cell, to generate at least 60 million reads with >80X mean coverage and >98% of target bases at ≥20X. Samples were sequenced in multiple batches as and when samples were received for diagnostic testing. The Illumina HiSeq FASTQ sequencing reads were demultiplexed and aligned to the reference (GRCh37/Hg19) by using BWA‐MEM (v0.7.12), converted to BAM format file and subjected to duplicate removal by using Picard (v1.129). GATK (v3.4‐46) was used for indel realignment, variant calling, and quality filtering.

### Variant annotation and filtering

**Figure 1:**
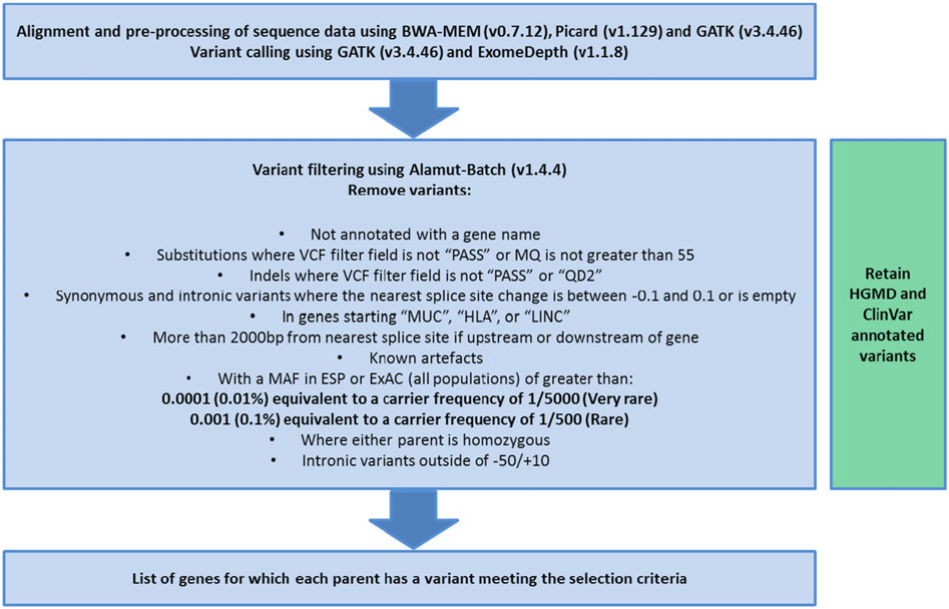
Bioinformatics pipeline. The filtering criteria are applied to generate a shortlist of genes in which both parents have a heterozygous variant meeting the criteria. Abbreviations: VCF (variant call format), MQ (mapping quality), QD2 (quality by depth), MUC (mucin antigen), HLA (human leukocyte antigen), LINC (LincRNA), MAF (minor allele frequency), ESP (Exome Sequencing Project http://evs.gs.washington.edu/EVS/), ExAC (Exome Aggregation Consortium http://exac.broadinstitute.org/), and dbSNP (NCBI short genetic variation database https://www.ncbi.nlm.nih.gov/projects/SNP/). [Colour figure can be viewed at wileyonlinelibrary.com]

Variants were annotated by using Alamut‐Batch (v1.4.4), a variant call format file was inputted and all SNVs and indels were annotated by using a range of different variant and genomic databases, including HGMD Professional.^8^ A bioinformatics pipeline was designed in‐house to identify shared genes where both parents had a heterozygous potentially pathogenic variant and to identify X‐linked recessive variants where appropriate (Figure 1). Variants with a MAF < 0.0001 (<0.01%) and 0.001 (<0.1%) in Exome aggregation consortium (ExAC http://exac.broadinstitute.org/) or the Exome variant server (EVS http://evs.gs.washington.edu/EVS/) were retained to produce a subset of very rare variants and rare variants. Variants were restricted to nonsynonymous variants, those affecting the conserved splice sites or those within −50/+10 base pairs of flanking exons predicted by Alamut‐Batch to affect splicing (5 tools were used: SpliceSiteFinder‐like, MaxEntScan, NNSplice [Fruitfly], GeneSplicer, and Human Splicing Finder). Variants annotated as Pathogenic in HGMD Pro (all cases) or ClinVar (since 2016) were retained regardless of other filtering criteria. Copy number variants were identified by using read depth analysis with a modified version of R software package ExomeDepth (v1.1.8)^9^ and comparing the test sample against reference samples. Autosomal recessive variants were identified where parents either shared the same heterozygous variant or had different heterozygous variants in the same gene. Potential X‐linked recessive variants in the mother were also shortlisted where only male pregnancies were affected. The bioinformatics pipeline is summarized in Figure 1.

### Selection of candidate variants

All genes on the variant shortlist were considered and initially reviewed via the Online Mendelian Inheritance in Man database (OMIM https://www.omim.org/) and PubMed (https://www.ncbi.nlm.nih.gov/pubmed/). Candidate variants in known disease‐causing genes were identified for further investigation by comparison with the fetal phenotype. Previous reports of a variant were determined by using HGMD professional, ClinVar (https://www.ncbi.nlm.nih.gov/clinvar/) and Locus‐specific databases. In silico tools were accessed via Alamut Visual (versions 2.7.2‐2.10) to predict pathogenicity of variants. Likely causative variants identified in this series were variants that were reported as likely pathogenic or pathogenic in the patient's clinical diagnostic report. We have reviewed all the variant classifications by using the recently published American College of Medical Genetics and Genomics (ACMG)/Association for Molecular Pathology guidelines.^10^ In cases where no likely diagnosis was identified, we undertook further analysis of a curated gene panel from PanelApp (https://bioinfo.extge.co.uk/crowdsourcing/PanelApp/).

### Confirmation of results

Possible disease‐causing variants identified in parental DNA samples were confirmed by PCR/Sanger Sequencing for SNVs and indels or using the QX200 droplet digital PCR system (Bio‐Rad, Hercules, California, USA) for CNVs. Fetal DNA samples were tested to establish co‐segregation of the variant(s) with disease. DNA from at least 1 affected fetus (or neonate) was available for testing for each couple. PCR primer and droplet digital PCR primer/probe sequences are available on request.

### RNA extraction and reverse transcription

For case 7, total RNA was extracted from PAXgene collection tubes (PreAnalytiX, Hombrechtikon, Switzerland) by using the PAXgene Blood miRNA Kit (Qiagen, Hilden, Germany). Complementary DNA (cDNA) was synthesized by using VILO SuperScript III RT‐PCR system (Thermo Fisher Scientific, Massachusetts, USA) and fragment/sequence analysis performed by using primers designed against *IFT122* exon 23 and 25. Products were visualized by electrophoresis on a 2% agarose gel.

## Results

**Figure 2:**
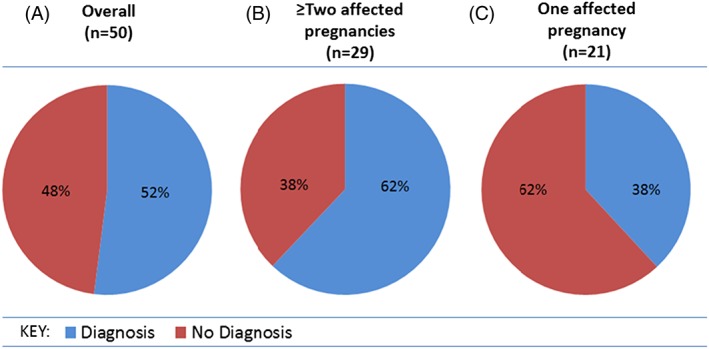
(A) The overall diagnostic yield in the 50 couples included in this audit. (b) The diagnostic yield for couples with 2 or more affected pregnancies. (C) The diagnostic yield for couples with a single affected pregnancy

**Figure 3:**
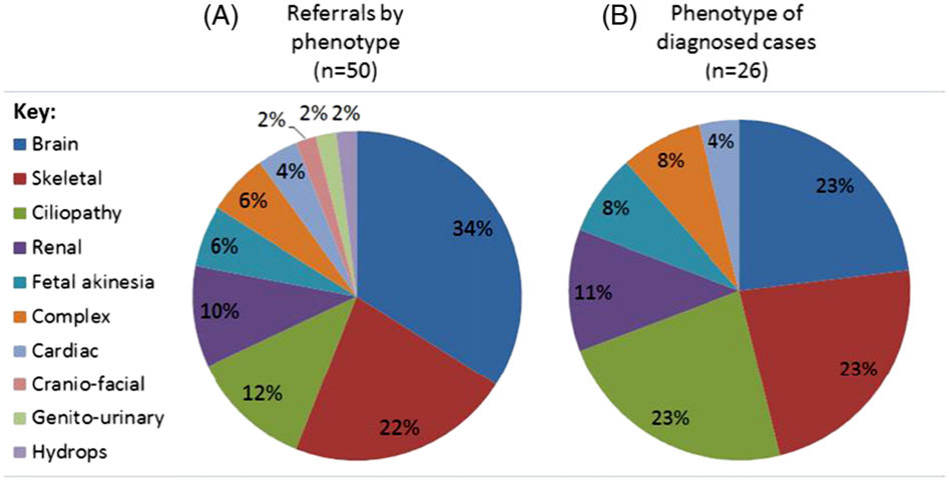
Pie charts to show the phenotypic spectrum for (A) all couples referred for testing by using this strategy and (B) the 26 couples with a genetic diagnosis

Heterozygous pathogenic or likely pathogenic variants in both partners were identified in 26/50 couples (52%), and where 2 or more fetuses were affected, a genetic diagnosis was obtained in 18/29 (62%) cases (Table 1 and Figures 2A and 2B). Of the 21 couples with only 1 affected pregnancy, a diagnosis was determined in 8/21 (38%, Figure 2C). No likely pathogenic variants were identified by exome sequencing in the remaining 24 cases. The phenotypic spectrum for the cases tested and diagnosed is illustrated in Figure 3. Cases 1 and 2 have been described previously.^5^ Variants classified as pathogenic or likely pathogenic were identified in 24 different genes (*ASPM*, *ATAD3A*, *ATRX*, *B3GLCT*, *BBS9*, *BBS10*, *CENPJ*, *DYNC2H1*, *ERCC5*, *ETFA*, *EXOSC3*, *FRAS1*, *GLE1* [n = 2], *IFT122*, *ITGA8*, *LRP4*, *MKS1*, *MRPS22*, *NEK9*, *POMGNT1*, *RYR1* [n = 2], *SASS6*, *TMEM67*, and *TRIP11*). Autosomal recessive inheritance was observed in 25 cases, and there was 1 family identified with an X‐linked recessive etiology (*ATRX* variant in case 11). The hemizygous *ATRX* variant was present in 2 affected male fetuses with arthrogryosis and talipes. Sex reversal was present in 1 fetus. Skewed X‐inactivation (100:0) was observed in their heterozygous mother. Variants in cases 13, 17, and 18 (compound heterozygous variants in *GLE1*, *BBS9*, and *DYNC2H1* genes) were reported as “likely pathogenic” by using the UK best practice guidelines (http://www.acgs.uk.com/media/774853/evaluation_and_reporting_of_sequence_variants_bpgs_june_2013_‐_finalpdf.pdf) prior to adoption of the ACMG guidelines. These have since been reviewed by using the ACMG guidelines that classifies them as variants of uncertain significance (Table S2) but are still felt to be “likely pathogenic” after clinical discussion. From a total of 41 identified variants, 9 of the variants identified were previously reported in the literature^11^, ^12^, ^13^, ^14^, ^15^, ^16^, ^17^, ^18^, ^19^, ^20^, ^21^, ^22^, ^23^ (Tables 1 and Table S1). Eleven couples with known consanguinity were included in the series, and a diagnosis was made in 6/11 (54%) of these couples. Homozygous variants were identified in 5 of these couples and compound heterozygous variants in the sixth.

**Table 1:** Summary of clinical information and results in the affected pregnancies where a genetic diagnosis was obtained

Case	Phenotype (HPO TERMS)	Affected Pregnancies	Consanguinity	Gene	HGVS Nomenclature	Variant Classification on Clinical Diagnostic Report	OMIM Diagnosis
01^5^	Hydrops fetalis, multiple joint contractures, pulmonary hypoplasia	≥2	N	*RYR1*	NM_000540.2 p.[(Ser3074Phe)];[?] c.[9221C>T];[14130‐2A>G]	Pathogenic	Fetal akinesia OMIM No.180901
02^5^	Arthrogryposis multiplex congenita, multiple pterygia	≥2	N	*GLE1*	NM_001003722.1 p.[(Arg569His)];[(Val617Met)] c.[1706G>A];[1849G>A]	p.(Arg569His)^11^ Likely pathogenic p.(Val617Met)^11^ Likely pathogenic	Lethal congenital contracture syndrome 1/lethal arthrogryposis with anterior horn cell disease OMIM no 603371
03	Hydrops fetalis, agenesis of corpus callosum, hypertrophic cardiomyopathy, pulmonary hypoplasia, low‐set ears, increased nuchal translucency, ventriculomegaly, wide anterior fontanel	≥2	N	*MRPS22*	NM_020191.2 p.[(Arg170His)];[?] c.[509G>A];[878+1G>T]	p.(Arg170His)^12^ Likely pathogenic c.878+1G>T Pathogenic	Combined oxidative phosphorylation deficiency 5 OMIM no 605810
04	Congenital microcephaly	1	N	*CENPJ*	NM_018451.4 p.[(Glu9Ter)];[(Gln971Ter)] c.[25G>T];[2911C>T]	Pathogenic	Autosomal recessive primary microcephaly 6 OMIM no 609279
07	Postaxial hand polydactyly, postaxial foot polydactyly, fibular aplasia, downslanted palpebral fissures, low‐set ears, cleft palate, intestinal malrotation, abnormality of pancreas morphology, absent tibia, preaxial foot polydactyly	≥2	N	*IFT122*	NM_052985.3 p.[(Ala688_Asp694delinsGlyVal)];[(?)] c.[2063_2082delinsGCGTG];[3039+4A>G]	Likely Pathogenic	Cranioectodermal dysplasia‐1 (CED1) OMIM no 218330
09	Absent hand (bilateral), absent toes, gastroschisis, intestinal malrotation, bilateral renal agenesis, ventriculomegaly, sex reversal	≥2	N	*LRP4*	NM_002334.3 p.[(Asp606Asn)];[(Gly629Glu)] c.[1816G>A];[1886G>A]	Likely Pathogenic	Cenani‐Lenz syndrome OMIM no 604270
11	Ambiguous genitalia, arthrogryposis multiplex congenita	≥2	N	*ATRX*	NM_000489.4 p.[(Asp2177Ala)];[0] c.[6530A>C];[0]	Likely Pathogenic	Mental retardation‐hypotonic facies syndrome, X‐linked OMIM no 309580
12	Hyperechogenic kidneys, polydactyly	≥2	N	*BBS10*	NM_024685.3 p.[(Cys91fs)];[(Cys91fs)] c.271[dup];[dup]	Pathogenic^13^, ^14^	Bardet‐Biedl syndrome 10 OMIM no 610148
13	Polyhydramnios, decreased fetal movement, arthrogryposis multiplex congenita, micrognathia, high palate, congenital hip dislocation, poor suck, focal seizures, increased serum lactate, hypoglycemia	≥2	N	*GLE1*	NM_001003722.1 p.[(Ser194Asn)];[(Arg670Leu)] c.[581G>A];[2009G>T]	Likely Pathogenic	Lethal congenital contracture syndrome 1/lethal arthrogryposis with anterior horn cell disease OMIM no 603371
14	Congenital microcephaly, agenesis of corpus callosum, cerebellar hypoplasia, 11 pairs of ribs	≥2	Y	*SASS6*	NM_194292.1 p.[(Glu412Gln)];[(Glu412Gln)] c.1235[A>G];[A>G]	Likely Pathogenic	Autosomal recessive primary microcephaly 14 OMIM no 609321
16	Arthrogryposis multiplex congenita, hydrops fetalis	≥2	N	*RYR1*	NM_000540.2 p.[(?)];[(Gly4782Arg)] c.[12013‐2A>G];[14344G>A]	p.(Gly4782Arg)^15^ Likely Pathogenic c.12013‐2A>G Pathogenic	Fetal akinesia OMIM no 180901
17	Hand polydactyly, foot polydactyly, multiple renal cysts , enlarged kidneys, hyperechogenic kidneys	1	Y	*BBS9*	NM_198428.2 p.[(Asn254Ser)];[(Gly306Glu)] c.[761A>G];[917G>A]	Likely Pathogenic	Bardet‐Biedl syndrome 9 OMIM no 615986
18	Short long bones, neonatal respiratory distress, short ribs, anterior rib cupping, thoracic hypoplasia, abnormality of the clavicle	1	N	*DYNC2H1*	NM_001080463.1 p.[(Thr1696Met)];[(Ser3281Asn)] c.[5087C>T];[9842G>A]	Likely Pathogenic	Short rib polydactyly type III OMIM no 603297
19	Bilateral renal agenesis, oligohydramnios, pulmonary hypoplasia, hypertrophic cardiomyopathy, anal atresia, aplasia of the uterus, aplasia/Hypoplasia of the fallopian tube	≥2	N	*FRAS1*	NM_025074.6 p.[?];[(Gly2004Ser)] c.[5530‐2A>C];[6010G>A]	c.5530‐2A>C Pathogenic p.(Gly2004Ser) Pathogenic	Fraser syndrome OMIM no 607830
20	Bilateral renal agenesis, oligohydramnios	≥2	Y	*ITGA8*	NM_003638.1 p.[(Val489fs)];[(Val489fs)] c.1466_1470[del];[del]	Pathogenic	Renal hypoplasia/aplasia 1 OMIM no 604063
21	Intrauterine growth retardation, hypertelorism, low‐set ears, megalencephaly, ventriculomegaly	≥2	N	*B3GLCT*	NM_194318.3 p.[?];[?] c.660+1[G>A];[G>A]	c.660+1G>A Pathogenic^16^, ^17^	Peters‐plus syndrome OMIM no 261540
27	Occipital meningoencephalocele, cystic renal dysplasia, polydactyly, 2‐3 toe syndactyly, low‐set ears, female external genitalia in individual with 46,XY karyotype, pulmonary hypoplasia	1	N	*MKS1*	NM_017777.3 p.[?];[?] c.1408‐34_1408‐6[del];[del]	c.1408‐34_1408‐6del^18^, ^19^ Pathogenic	Meckel syndrome 1 OMIM no 249000
28	Ventriculomegaly	≥2	N	*POMGNT1*	NM_001243766.1 p.[(Arg497Gln)];[(Arg497Gln)] c.1490[G>A];[G>A]	Likely Pathogenic	Muscular dystrophy‐dystroglycanopathy type A3 OMIM No. 253280
29	Arthrogryposis multiplex congenita	1	Y	*ERCC5*	NM_000123.3 p.[(Gln622Ter)];[(Gln622Ter)] c.1864[C>T];[C>T]	Pathogenic	Cerebrooculofacioskeletal syndrome 3 OMIM no 616570
31	Cerebellar hypoplasia	1	N	*EXOSC3*	NM_016042.3 p.[(Gly31Ala)];[(Gly31Ala)] c.92[G>C];[G>C]	p.(Gly31Ala)^20^, ^21^, ^22^ Pathogenic	Pontocerebellar hypoplasia, type 1B OMIM no 614678
34	Bilateral renal dysplasia, multiple renal cysts	≥2	N	*ETFA*	NM_000126.3 p.[(Arg223Ter)];[?] c.[667C>T];[(882+1_883‐1)_(963+1_964‐1)del]	p.(Arg223Ter) Pathogenic Exon 11 deletion Likely Pathogenic	Glutaric acidemia IIA OMIM no 231680
37	Tetraamelia, kyphoscoliosis, absent septum pellucidum, abnormal cortical gyration	≥2	N	*TRIP11*	NM_004239.4 p.[(Arg225Ter)];[(Val671fs)] c.[673C>T];[2010del]	Pathogenic	Achondrogenesis, type IA OMIM no 200600
39	Arthrogryposis multiplex congenita, fetal akinesia sequence	≥2	Y	*NEK9*	NM_033116.5 p.[(Glu500fs)];[(Glu500fs)] c.1498[del];[del]	Pathogenic	Lethal contracture syndrome type 10 OMIM no 617022
41	Hypertrophic cardiomyopathy, cerebellar hypoplasia, cryptorchidism, opacification of the corneal stroma, neonatal asphyxia, neonatal hypotonia	≥2	N	*ATAD3A*	NM_001170535.1 p.[(Phe50Leu)];[?] c.[150C>G];[(282+1_283‐1)_(444+1_445‐1)del]	p.(Phe50Leu) Likely pathogenic Exon 3‐4 deletion Likely pathogenic	Harel‐Yoon syndrome OMIM no 617183
47	Cerebellar hypoplasia, ventriculomegaly, enlarged kidneys, decreased fetal movements, abnormality of the amniotic fluid	1	N	*TMEM67*	NM_153704.5 p.[(Arg74Ter)];[(Trp346Cys)] c.[220C>T];[1038G>T]	p.(Arg74Ter) Pathogenic p.(Trp346Cys) Likely pathogenic	Meckel syndrome type 3 OMIM no 607361
48	Microcephaly, flat forehead, proptosis	1	Y	*ASPM*	NM_018136.4 p.[(Gln421fs)];[(Gln421fs)] c.1260_1266[del];[del]	p.(Gln421fs)^23^ Pathogenic	Primary Microcephaly type 5 OMIM no 608716

Cases 1 and 2 have been described previously.^5^

†For ACMG classification see Table S1. The reference is included for variants previously reported in the literature.

Copy number variants were identified as likely disease‐causing in 2 families; an *ETFA* exon 11 deletion (case 34) and an *ATAD3A* exon 3 to 4 deletion (case 41). Four couples had causative variants in recently discovered genes where there is only 1 publication or <3 cases previously reported: *ATAD3A*, *ITGA8*, *NEK9*, and *SASS6*. Well‐described founder mutations in *GLE1*, *BBS10*, *B3GLCT*, *MKS1*, and *EXOSC3* were identified in 5 families. The *MKS1* variant was identified through a gene panel analysis as the MAF is greater than 0.001, and the nomenclature recorded in HGMD Pro for this founder mutation is discordant with the Alamut annotation.

**Figure 4:**
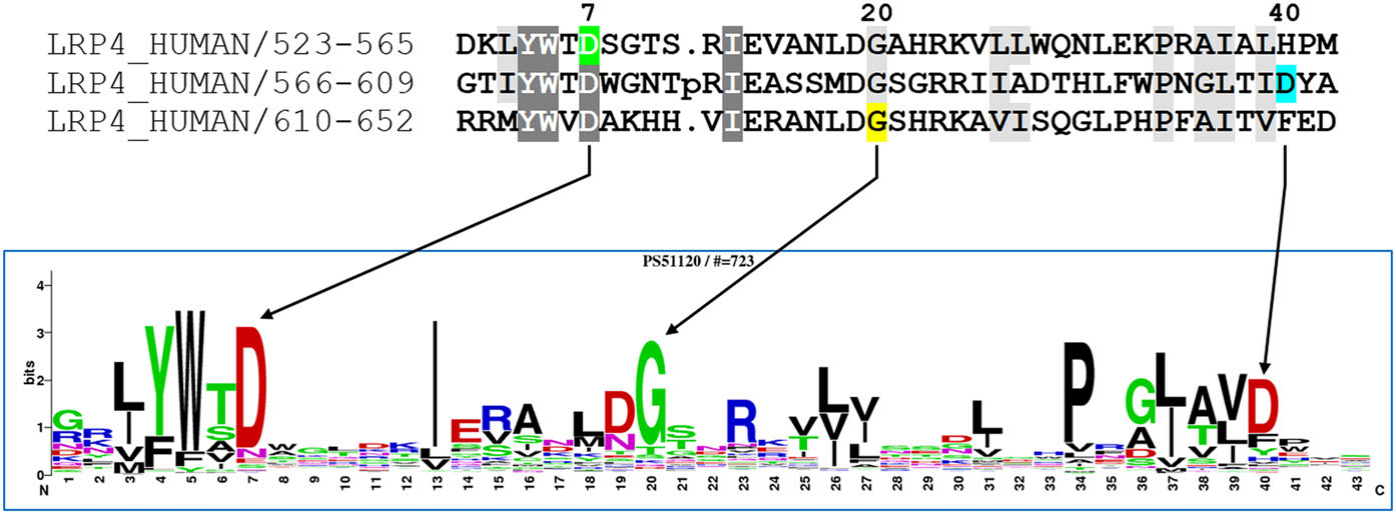
The upper panel shows an alignment of the YWTD repeats 2 to 4 of human LRP4 from PROSITE entry PS51120 (gaps removed), highlighting the variants identified in case 9, p.(Gly629Glu) (yellow) and p.(Asp606Asn) (blue) and the previously reported p.(Asp529Asn) (green). Representation of the sequence logo below indicates that the wild‐type residues are the most commonly seen, whereas the variant residues are either absent or present at a low frequency (≤1.5%)

Rare *LRP4* missense variants, p.(Asp606Asn) and p.(Gly629Glu), were identified in case 9. Pathogenic variants in this gene are a known cause of Cenani‐Lenz syndrome, which is characterized by syndactyly/oligodactyly and kidney abnormalities.^24^ Sanger sequencing confirmed that the affected fetus was compound heterozygous for the missense variants. In silico tools supported pathogenicity of both missense variants, which lie within conserved LDLR receptor class B repeats 3 and 4, respectively, of LRP4. The PROSITE database entry for the LDLR receptor class B repeat (entry PS51120) contains 683 repeats from 62 different proteins; alignment of LRP4 repeats 3 and 4 with the consensus sequence shows that the p.(Asp606Asn) and p.(Gly629Glu) variants lie at positions 40 and 20 of the repeat motif, respectively (Figure 4). Inspection of the PROSITE sequence logo indicated that aspartic acid and glycine are the most commonly observed amino acids at these positions (at frequencies of ~68% and ~79%, respectively), whereas the variant residues were absent or present at very low frequencies (≤1.5%). By comparison, a previously reported pathogenic *LRP4* missense variant, p.(Asp529Asn),^24^ occurs at position 7 of the motif within the second LDLR receptor class B repeat, and although aspartic acid is most commonly seen at this position (~81% frequency in the sequence logo), the variant residue asparagine is observed in 41/683 sequences in the PROSITE entry (6%). This suggests that the p.(Asp529Asn) is more likely to be tolerated than the variants identified in this couple and thus have a milder effect on structure and function of the repeat resulting in a less severe phenotype.

**Figure 5:**
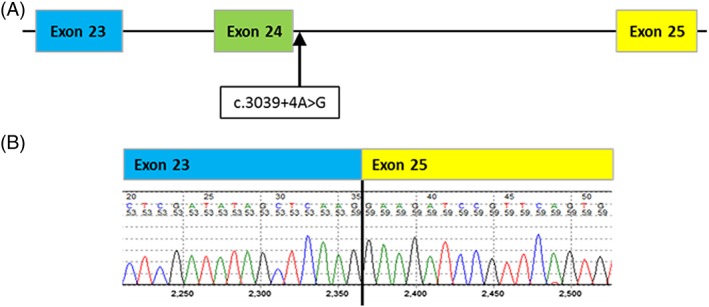
(A) Location of the *IFT122* variant, NC_000003.11(NM_052985.3)c.3039+4A>G, in intron 24. (b) Sequence electropherogram of the *IFT122* RT‐PCR product demonstrating the effect of this splicing variant in the maternal RNA. The primers used were designed over the suspected breakpoint (exon23‐exon25 boundary) so only the variant allele was amplified

Messenger RNA analysis was undertaken for a couple with novel *IFT122* variants (case 7). The paternal variant was a large insertion‐deletion that results in the net loss of 5 amino acids in exon 17 of the *IFT122* gene and is predicted to result in significant changes to the arrangement of secondary structure motifs. The maternal variant was a base substitution, c.3039+4A>G, in intron 24 (Figure 5A), and the splice prediction tools suggest a reduction in the strength of the 5′ acceptor site of between 9.1% and 30%. Sanger sequencing of PCR products generated by using primers designed to amplify from exon 23 to exon 25 of cDNA from the carrier mother is consistent with exon 24 skipping (Figure 5B).

An additional, or unsolicited, finding was identified in 1 consanguineous couple who are both heterozygous carriers of a pathogenic frameshift variant, c.3503_3504del, p.(Leu1168fs) in the *GNPTAB* (NM_024312.4) gene. This is a previously reported founder mutation, and recessive variants are a known cause of Mucolipidosis II alpha/beta.^25^, ^26^

## Discussion

Using the parental autosomal recessive strategy, a potential genetic diagnosis was established in 26/50 couples, giving a diagnostic yield of 52%. This increases to 18/29 (62%) in those couples who have had 2 or more pregnancies affected with lethal or prenatal‐onset disorders. Potentially pathogenic variants were identified in 24 different genes, consistent with the known genetic heterogeneity of these rare disorders. By using the parental DNA for exome sequencing, we can conserve the precious DNA from the affected pregnancies; using a small quantity for directed variant confirmation.

In most cases, the reported phenotypes in these families were consistent with that reported in the literature. Some cases identified a diagnosis that would not usually be readily identified in the prenatal period. A diagnosis of Peters‐plus syndrome was made in family 21 after the identification of the founder mutation, c.660+1G>A, in the *B3GLCT* gene (formerly known as *B3GALTL*). A diagnosis of Peters‐plus syndrome is often made postnatally; prenatal diagnosis has been reported but is generally more difficult due to variable and nonspecific findings.^27^ Severe hydrocephalus was the only indication of this diagnosis. Similarly, the fetuses with the *POMGNT1* homozygous missense variant had presented with ventriculomegaly, an early feature of muscle‐eye‐brain disease (case 28).

Other cases confirmed an extension of a known phenotype or evidence of a genotype‐phenotype correlation. *LRP4* missense variants, p.(Asp606Asn) and p.(Gly629Glu), were identified in case 9. Pathogenic missense or splicing *LRP4* variants are a known cause of Cenani‐Lenz syndrome, which is characterized by a less severe phenotype than was seen in our patient, of syndactyly/oligodactyly and kidney abnormalities.^24^ A recent report of truncating variants causing a severe lethal prenatal form^28^, ^29^ is consistent with Cenani‐Lenz syndrome being the result of hypomorphic variants. This suggested that some missense variants could have a severe impact on protein function and result in a severe lethal prenatal phenotype. The p.Asp606 and p.Gly629 residues are located within the highly conserved LRP4 YWTD motif. Variant residues at these positions are either absent or rarely observed in this sequence and are unlikely to be tolerated within the repeat region. A missense variant, p.(Asp529Asn), reported in a patient with Cenani‐Lenz syndrome^24^ is observed at a frequency of ~6%, and although this variant is known to be pathogenic, it is clearly compatible with life. We predict that both p.(Asp606Asn) and p.(Gly629Glu) may have a more severe effect, consistent with observed lethality in the case of the fetuses with compound heterozygosity for these 2 variants.

Similarly, recessive pathogenic *IFT122* variants are known to cause Cranioectodermal dysplasia 1,^30^ which is a nonlethal condition where patients present with craniofacial, skeletal, and ectodermal abnormalities. One couple has been reported with recurrent pregnancy losses and compound heterozygosity for 2 *IFT122* mutations (a missense and frameshift).^31^ More recently, *IFT122* biallelic variants, a missense and a frameshift, were identified in a male infant born prematurely at 31/40 weeks of gestation with a typical phenotype of short‐rib polydactyly type IV.^32^ We found compound heterozygous *IFT122* variants in 1 family (case 7) with a skeletal dysplasia. The paternally inherited variant was a large insertion‐deletion in exon 17 of *IFT122*. In‐frame insertions and deletions in nonrepetitive regions of greater than 1 amino acid are considered more likely to disrupt protein function than a missense variant alone; the larger the deletion or insertion, the more likely it is to be pathogenic.^10^ These large in‐frame insertions/deletions are rare in population databases reflecting the fact that they generally have deleterious effects on protein structure and folding, particularly when they overlap or occur within regions of secondary structure. In silico tools predicted aberrant splicing due to a maternal variant, c.3039+4A>G in intron 24, and messenger RNA studies supported that prediction with skipping of exon 24 observed in a maternal blood sample. Thus, evidence suggests that these 2 variants are likely to be pathogenic and provide a plausible explanation of the phenotype seen in their affected offspring. This case also illustrates the extended spectrum of phenotypes associated with known disorders identified due to exome sequencing.

In 3 families, a “clinical exome” test was performed where a subset of the exome was sequenced (~6110 genes) and a genetic diagnosis was established in 2 of the 3 families. We recommend that WES is undertaken for the undiagnosed couple because in our cohort, 3 of the 26 diagnoses made by using the whole exome capture would have missed using this “clinical exome” approach targeting 6,110 genes associated with human diseases (genes known at the time of the capture design in 2013).

A novel *SASS6* variant, p.(Glu412Gly), was identified in both partners of a couple who had 2 pregnancies with primary microcephaly (case 14). This gene has recently been identified by homozygosity mapping using WES in a large consanguineous Pakistani family with multiple family members affected with microcephaly.^33^ Homozygous *NEK9* variants were identified in family 39 who were known to be consanguineous and had 2 affected pregnancies with arthrogryposis and fetal akinesia sequence. In 2016, this gene was linked to lethal contracture syndrome type 10 in 2 Irish traveler families.^34^ Couple 41 suffered 2 pregnancy losses at birth; both babies had no respiratory effort and congenital hypertrophic cardiomyopathy. Both affected offspring were compound heterozygous for an *ATAD3A* novel missense variant and a deletion of exons 3 and 4. Monoallelic and biallelic variants have recently been reported to cause Harel‐Yoon syndrome, which is characterized by global developmental delay, hypotonia, optic atrophy, axonal neuropathy, and hypertrophic cardiomyopathy.^35^ Compared with the whole exome test, the clinical exome is cheaper, yields a shorter list of variants for analysis, and may give higher coverage across known disease genes but, as demonstrated in these 3 cases, has the disadvantage of not including the most recently discovered, or not yet identified, disease genes.

Using this strategy, only shared genes where both parents harbor a rare variant are included in the analysis; this limits the risk of detection of a variant in later‐onset dominant disorders, such as cancer predisposition genes. However, this approach does not exclude the possibility of revealing carrier status for other autosomal recessive disorders and this risk is increased in consanguineous couples. In one of the consanguineous couples in this case series, we identified an additional, unrelated finding where both parents were heterozygous for a founder mutation in the *GNPTAB* gene. As this is a clearly pathogenic variant, we discussed it with the referring clinician who agreed to disclose the finding due to the 1 in 4 risk for this couple having a child affected with Mucolipidosis II alpha/beta in addition to the 1 in 4 risk of inheriting the variants causative of the recessive condition that had already presented. We note that if there had been sufficient DNA available for a trio analysis, this additional finding might not have been detected. The couple found the information on the incidental finding useful and informative.

There are multiple reasons why likely disease‐causing variants were not identified in 24 of the 50 families reported here. It is reasonable to assume an autosomal recessive pattern of inheritance in a family with multiple affected offspring or in a consanguineous pairing, but a different mode of inheritance cannot be excluded. Recent case reports describe families with multiple affected children with megacystis microcolon intestinal hypoperistalsis syndrome, due to de novo disease‐causing variants in the *ACTG2* gene, suggestive of gonadal mosaicism.^36^ Similarly, diagnostic exome sequencing in 13 families with known consanguinity indicated that over 38% of positive results were not autosomal recessive.^37^ One of the 11 couples tested who were known to be consanguineous each had a different missense variant in the *BBS9* gene that was compound heterozygous in their affected fetus. Other explanations for not identifying a diagnosis include that the disorder is not monogenic, the pathogenic variant may be noncoding, the pathogenic variant may be a structural variant, the disease gene may not yet be associated with a disease phenotype, the causative gene may be poorly covered by the capture, or pathogenic variants could have been filtered out by the bioinformatic strategy/software. Interpretation of variants is based on information that is currently available, and this is likely to change as knowledge increases. One of the advantages of WES is that the data can be reanalyzed in the future as new disease genes are annotated.^2^

## Conclusion

In summary, we demonstrate that parental exome sequencing is a powerful tool to diagnose lethal or prenatal‐onset recessive fetal disorders. For those couples where insufficient fetal DNA is available for exome sequencing, this strategy provides the opportunity of a genetic diagnosis to provide preimplantation or prenatal diagnosis for future pregnancies.

## Guarantor Statement

SE is the guarantor of this work and, as such, had full access to all the data in the study and takes responsibility for the integrity of the data and the accuracy of the data analysis.

**What's already known about this topic?**

- Exome sequencing is used routinely for postnatal diagnosis of rare disorders with a diagnostic yield of 20 to 40%.
- Insufficient quantity or quality of DNA restricts the use of exome sequencing for diagnosing lethal fetal disorders.
- Couples are counselled for a likely 25% recurrence risk, but without a genetic diagnosis, no molecular prenatal test is possible.
- A parental exome sequencing strategy has been applied successfully in a small number of couples.

**What does this study add?**

- We show that exome sequencing of parental DNA samples is an effective way to diagnose lethal or prenatal‐onset disorders with a diagnostic yield of 52% in an audit of 50 consecutive cases.
- Testing can be carried out in the prenatal period to guide management of an ongoing pregnancy or for use in subsequent pregnancies to allow couples the option of a prenatal or preimplantation genetic test.

## Supporting information

Table S1. This table summarizes the clinical phenotype in the affected pregnancies in the 24 families where a genetic diagnosis was not identified.

Table S2. This table details the results from the 26 cases where a diagnosis was made. The variant is described at the genomic, cDNA, and protein level and shows the ACMG codes and level of evidence used for classification. ^†^Variants were reported on the clinical report as likely pathogenic prior to implementation of the ACMG guidelines and have been reviewed following adoption. The variants were identified by a gene‐agnostic approach and following discussion with referring clinicians were felt to be likely causative in these families

## References

[1] Yang Y , Muzny DM , Reid JG , *et al* Clinical whole‐exome sequencing for the diagnosis of mendelian disorders. N Engl J Med 2013 Oct 17;369(16):1502–1511. 24088041 10.1056/NEJMoa1306555 PMC4211433

[2] Yang Y , Muzny DM , Xia F , *et al* Molecular findings among patients referred for clinical whole‐exome sequencing. JAMA 2014 Nov 12;312(18):1870–1879. 25326635 10.1001/jama.2014.14601 PMC4326249

[3] xLarge‐scale discovery of novel genetic causes of developmental disorders. Nature 2015 Mar 12;519(7542):223–228. 25533962 10.1038/nature14135 PMC5955210

[4] Reuter MS , Tawamie H , Buchert R , *et al* Diagnostic yield and novel candidate genes by exome sequencing in 152 consanguineous families with neurodevelopmental disorders. JAMA Psychiat 2017 Mar 01;74(3):293–299. 10.1001/jamapsychiatry.2016.3798 28097321

[5] Ellard S , Kivuva E , Turnpenny P , *et al* An exome sequencing strategy to diagnose lethal autosomal recessive disorders. Eur J Hum Genet 2015 Mar;23(3):401–404. 24961629 10.1038/ejhg.2014.120 PMC4205099

[6] Drury S , Williams H , Trump N , *et al* Exome sequencing for prenatal diagnosis of fetuses with sonographic abnormalities. Prenat Diagn 2015 Oct;35(10):1010–1017. 26275891 10.1002/pd.4675

[7] Shamseldin HE , Kurdi W , Almusafri F , *et al* Molecular autopsy in maternal‐fetal medicine. Genet Med 2017; Jul 27. 10.1038/gim.2017.111 28749478

[8] Stenson PD , Mort M , Ball EV , *et al* The Human Gene Mutation Database: 2008 update. Genome Med 2009;1(13):13. 19348700 10.1186/gm13 PMC2651586

[9] Plagnol V , Curtis J , Epstein M , *et al* A robust model for read count data in exome sequencing experiments and implications for copy number variant calling. Bioinformatics 2012 Nov 1;28(21):2747–2754. 22942019 10.1093/bioinformatics/bts526 PMC3476336

[10] Richards S , Aziz N , Bale S , *et al* Standards and guidelines for the interpretation of sequence variants: a joint consensus recommendation of the American College of Medical Genetics and Genomics and the Association for Molecular Pathology. Genet Med 2015 Mar;5:405–423. 10.1038/gim.2015.30 PMC4544753 25741868

[11] Nousiainen HO , Kestila M , Pakkasjarvi N , *et al* Mutations in mRNA export mediator GLE1 result in a fetal motoneuron disease. Nat Genet 2008 Feb;40(2):155–157. 18204449 10.1038/ng.2007.65 PMC2684619

[12] Saada A , Shaag A , Arnon S , *et al* Antenatal mitochondrial disease caused by mitochondrial ribosomal protein (MRPS22) mutation. J Med Genet 2007 Dec;44(12):784–786. 17873122 10.1136/jmg.2007.053116 PMC2652816

[13] Stoetzel C , Laurier V , Davis EE , *et al* BBS10 encodes a vertebrate‐specific chaperonin‐like protein and is a major BBS locus. Nat Genet 2006 May;38(5):521–524. 16582908 10.1038/ng1771

[14] Sathya Priya C , Sen P , Umashankar V , *et al* Mutation spectrum in BBS genes guided by homozygosity mapping in an Indian cohort. Clin Genet 2015 Feb;87(2):161–166. 24400638 10.1111/cge.12342

[15] Westerfield LE , Stover SR , Mathur VS , *et al* Reproductive genetic counseling challenges associated with diagnostic exome sequencing in a large academic private reproductive genetic counseling practice. Prenat Diagn 2015 Oct;35(10):1022–1029. 26275793 10.1002/pd.4674

[16] Lesnik Oberstein SA , Kriek M , White SJ , *et al* Peters Plus syndrome is caused by mutations in B3GALTL, a putative glycosyltransferase. Am J Hum Genet 2006 Sep;79(3):562–566. 16909395 10.1086/507567 PMC1559553

[17] Schoner K , Kohlhase J , Muller AM , *et al* Hydrocephalus, agenesis of the corpus callosum, and cleft lip/palate represent frequent associations in fetuses with Peters' plus syndrome and B3GALTL mutations. Fetal PPS phenotypes, expanded by Dandy Walker cyst and encephalocele. Prenat Diagn 2013 Jan;33(1):75–80. 23161355 10.1002/pd.4012

[18] Kyttala M , Tallila J , Salonen R , *et al* MKS1, encoding a component of the flagellar apparatus basal body proteome, is mutated in Meckel syndrome. Nat Genet 2006 Feb;38(2):155–157. 16415886 10.1038/ng1714

[19] Szymanska K , Berry I , Logan CV , *et al* Founder mutations and genotype‐phenotype correlations in Meckel‐Gruber syndrome and associated ciliopathies. Cilia 2012 Oct 01;1(1):18. 23351400 10.1186/2046-2530-1-18 PMC3579735

[20] Wan J , Yourshaw M , Mamsa H , *et al* Mutations in the RNA exosome component gene EXOSC3 cause pontocerebellar hypoplasia and spinal motor neuron degeneration. Nat Genet 2012 Apr 29;44(6):704–708. 22544365 10.1038/ng.2254 PMC3366034

[21] Eggens VR , Barth PG , Niermeijer JM , *et al* EXOSC3 mutations in pontocerebellar hypoplasia type 1: novel mutations and genotype‐phenotype correlations. Orphanet J Rare Dis 2014 Feb 13;9:23. 24524299 10.1186/1750-1172-9-23 PMC3928094

[22] Schwabova J , Brozkova DS , Petrak B , *et al* Homozygous EXOSC3 mutation c.92G‐‐>C, p.G31A is a founder mutation causing severe pontocerebellar hypoplasia type 1 among the Czech Roma. J Neurogenet 2013 Dec;27(4):163–169. 23883322 10.3109/01677063.2013.814651

[23] Bond J , Roberts E , Mochida GH , *et al* ASPM is a major determinant of cerebral cortical size. Nat Genet 2002 Oct;32(2):316–320. 12355089 10.1038/ng995

[24] Li Y , Pawlik B , Elcioglu N , *et al* LRP4 mutations alter Wnt/beta‐catenin signaling and cause limb and kidney malformations in Cenani‐Lenz syndrome. Am J Hum Genet 2010 May 14;86(5):696–706. 20381006 10.1016/j.ajhg.2010.03.004 PMC2869043

[25] Kudo M , Brem MS , Canfield WM . Mucolipidosis II (I‐cell disease) and mucolipidosis IIIA (classical pseudo‐hurler polydystrophy) are caused by mutations in the GlcNAc‐phosphotransferase alpha/beta‐subunits precursor gene. Am J Hum Genet 2006 Mar;78(3):451–463. 16465621 10.1086/500849 PMC1380288

[26] Coutinho MF , Encarnacao M , Gomes R , *et al* Origin and spread of a common deletion causing mucolipidosis type II: insights from patterns of haplotypic diversity. Clin Genet 2011 Sep;80(3):273–280. 20880125 10.1111/j.1399-0004.2010.01539.x

[27] Gupta N , Kaul A , Kabra M . Prenatal diagnosis of fetal Peters' plus syndrome: a case report. Case Rep Genet 2013;2013: 364529. 10.1155/2013/364529 PMC3745879 23984120

[28] Kariminejad A , Stollfuss B , Li Y , *et al* Severe Cenani‐Lenz syndrome caused by loss of LRP4 function. Am J Med Genet A 2013 Jun;161A(6):1475–1479. 23636941 10.1002/ajmg.a.35920

[29] Lindy AS , Bupp CP , McGee SJ , *et al* Truncating mutations in LRP4 lead to a prenatal lethal form of Cenani‐Lenz syndrome. Am J Med Genet A 2014 Sep;164A(9):2391–2397. 24924585 10.1002/ajmg.a.36647

[30] Walczak‐Sztulpa J , Eggenschwiler J , Osborn D , *et al* Cranioectodermal Dysplasia, Sensenbrenner syndrome, is a ciliopathy caused by mutations in the IFT122 gene. Am J Hum Genet 2010 Jun 11;86(6):949–956. 20493458 10.1016/j.ajhg.2010.04.012 PMC3032067

[31] Tsurusaki Y , Yonezawa R , Furuya M , *et al* Whole exome sequencing revealed biallelic IFT122 mutations in a family with CED1 and recurrent pregnancy loss. Clin Genet 2014 Jun;85(6):592–594. 23826986 10.1111/cge.12215

[32] Silveira KC , Moreno CA , Cavalcanti DP . Beemer‐Langer syndrome is a ciliopathy due to biallelic mutations in IFT122. Am J Med Genet A 2017 May;173(5):1186–1189. 28370949 10.1002/ajmg.a.38157

[33] Khan MA , Rupp VM , Orpinell M , *et al* A missense mutation in the PISA domain of HsSAS‐6 causes autosomal recessive primary microcephaly in a large consanguineous Pakistani family. Hum Mol Genet 2014 Nov 15;23(22):5940–5949. 24951542 10.1093/hmg/ddu318

[34] Casey JP , Brennan K , Scheidel N , *et al* Recessive NEK9 mutation causes a lethal skeletal dysplasia with evidence of cell cycle and ciliary defects. Hum Mol Genet 2016 May 01;25(9):1824–1835. 26908619 10.1093/hmg/ddw054

[35] Harel T , Yoon WH , Garone C , *et al* Recurrent de novo and biallelic variation of ATAD3A, encoding a mitochondrial membrane protein, results in distinct neurological syndromes. Am J Hum Genet 2016 Oct 06;99(4):831–845. 27640307 10.1016/j.ajhg.2016.08.007 PMC5065660

[36] Tuzovic L , Tang S , Miller RS , *et al* New insights into the genetics of fetal megacystis: ACTG2 mutations, encoding gamma‐2 smooth muscle actin in megacystis microcolon intestinal hypoperistalsis syndrome (Berdon syndrome). Fetal Diagn Ther 2015;38(4):296–306. 25998219 10.1159/000381638

[37] Powis Z , Farwell KD , Alamillo CL , *et al* Diagnostic exome sequencing for patients with a family history of consanguinity: over 38% of positive results are not autosomal recessive pattern. J Hum Genet 2016 Feb;61(2):173–175. 26490185 10.1038/jhg.2015.125

